# Recent Developments in the Medicinal Applications of Silver-NHC Complexes and Imidazolium Salts

**DOI:** 10.3390/molecules22081263

**Published:** 2017-07-27

**Authors:** Nicholas A. Johnson, Marie R. Southerland, Wiley J. Youngs

**Affiliations:** 1 Department of Chemistry, 401 College Ave., Ashland University, Ashland, OH 44805, USA; njohnso9@ashland.edu; 2 Department of Chemistry, University of Akron, Akron, OH 44325, USA; mrs184@zips.uakron.edu

**Keywords:** silver, NHC, imidazolium salt, antibacterial, antitumor, chemotherapeutic

## Abstract

Because of their great structural diversity and multitude of chemical properties, N-heterocyclic carbenes (NHCs) have been utilized in a variety of capacities. Most recently, NHCs have been utilized as carrier molecules for many transition metals in medicinal chemistry. Specifically, Ag(I)-NHCs have been investigated as potent antibacterial agents and chemotherapeutics and have shown great efficacy in both in vitro and in vivo studies. Ag(I)-NHC compounds have been shown to be effective against a wide range of both Gram-positive and Gram-negative bacterial strains. Many compounds have also shown great efficacy as antitumor agents demonstrating comparable or better antitumor activity than standard chemotherapeutics such as cisplatin and 5-fluorouracil. While these compounds have shown great promise, clinical use has remained an unattained goal. Current research has been focused upon synthesis of novel Ag(I)-NHC compounds and further investigations of their antibacterial and antitumor activity. This review will focus on recent advances of Ag(I)-NHCs in medicinal applications.

## 1. Introduction

Beginning with the successful isolation of the first stable *N*-heterocyclic carbene (NHC) by Arduengo in 1991 [1], NHCs have become a staple in research laboratories throughout the world. NHCs started as a mere laboratory curiosity but have evolved into a versatile organic ligand that has been used in a variety of capacities [2]. This is mainly due to their unparalleled structural diversity, as NHCs can be substituted with a variety of functional groups at various positions on the NHC core. They are also relatively easy synthetic targets. Due to the strong σ-donor capabilities as well as the ability to participate in π-back bonding, NHCs offer the synthetic versatility to bind both hard and soft metals [3,4].

Metal-NHC complexes have been primarily used in catalytic chemistry [5], however, more recently NHC ligands have been used as carrier molecules for metals in biological applications. A multitude of transition metals have been used in conjunction with NHCs including copper, silver, gold, platinum, palladium, ruthenium and others. The reported number of NHC-metal complexes is continually growing and several excellent reviews on this topic have previously been published [6,7,8,9,10,11,12,13,14].

While the majority of organometallic pharmaceutical research has been focused upon platinum and gold, the medicinal applications of silver are well documented [15]. Silver is relatively non-toxic to humans, although prolonged exposure can cause rare pigmentation of the skin and eyes [16]. While the mechanism of action of silver based pharmaceuticals has not been fully elucidated, it seems to involve the release of Ag(I) that can enter the cell membrane and disrupt its function [17]. Therefore, employing ligands that can strongly coordinate to the active Ag(I) ions is essential. NHC ligands have been utilized to form more stable Ag(I) compounds because of relatively strong silver-carbon bond and the NHC core provides the ability to be tailored for many different applications. This review will focus on the recent and most relevant advances in the medicinal applications of Ag(I)-NHC complexes including antibacterial and anticancer activity.

## 2. Antibacterial Activity of Silver NHC Complexes

**Figure 1:**
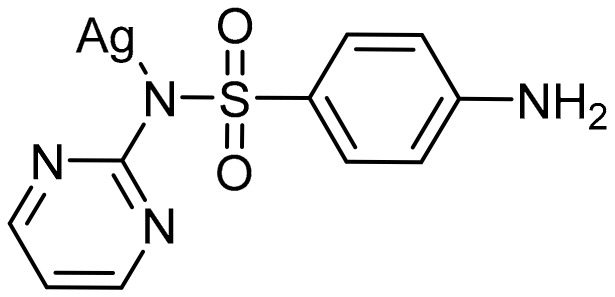
Structure of silver sulfadiazine.

The antibacterial properties of silver have been used throughout history [18,19]. The use of silver even predates modern civilizations, as early records describe water being stored in silver urns to prevent bacterial growth [20]. As early as 1840 silver nitrate was used to prevent infection in patients with severe burns [18]. Although silver was shown to be effective, with the discovery of penicillin and the production of many other penicillin-based antibiotics, the use of silver-based antimicrobials was significantly decreased. Soon after the wide spread use of penicillin and its derivatives, several bacterial strains that were resistant to these antibiotics emerged, including *Staphylococcus aureus* and methicillin-resistant *Staphylococcus aureus* (MRSA). This led to the resurgence of silver-based antimicrobial research, begun by Moyer in the 1960s [21] and quickly followed by the development by Fox of silver sulfadiazine, also known as Silvadene^®^ (Figure 1) [22]. Silver therapeutics have been used in burn wards and treatment centers since 1968 and continue to be a first line of defense against infections. Although the antimicrobial properties of silver are well known, the mechanism of action has not been fully elucidated. While many reports and reviews indicate that Ag(I) is the bioactive species, studies suggest that Ag(I) ions kill organisms in a variety of ways, contributing to the lack of silver resistance that is observed [20,23,24].

**Figure 2:**
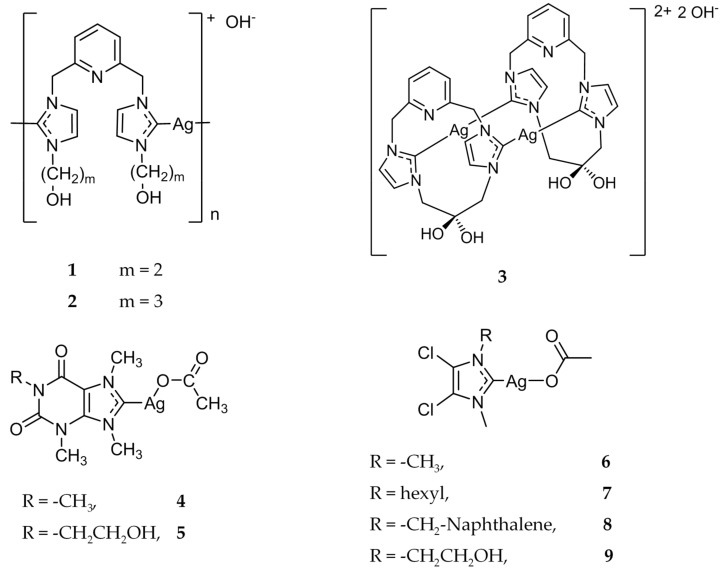
Structures of Ag(I)-NHC complexes synthesized by Youngs and coworkers [26,27,28,29,34].

This long history of silver’s uses in the medicinal field encouraged the creation of the first Ag(I)-NHC complex with antimicrobial properties [25]. In 2004, Youngs and coworkers synthesized several pincer Ag(I)-carbene complexes (Figure 2) [26], and tested their antimicrobial properties against *Escherichia coli (E. coli*), *Staphylococcus aureus* (*S. aureus*), and *Pseudomonas aeruginosa* (*P. aeruginosa)*. The minimum inhibitory concentration (MIC) of compounds **1** and **2** (Figure 2) were evaluated and showed better bacteriostatic activity than AgNO_3_, even at a much lower concentration. The success of this first Ag(I)-NHC antimicrobial was quickly followed by the development of the dinuclear species, **3** (Figure 2) [27]. The aqueous form of **3** was determined to be two times less effective as an antibiotic when compared to AgNO_3_. However, the antimicrobial activity of **3** was able to be enhanced via encapsulation in Tecophilic, a family of hydrophilic polyether-based thermoplastic aliphatic polyurethanes that are capable of being electrospun. The prepared nanofibers were again tested against *E. coli*, *S. aureus*, and *P. aeruginosa* for over a week with daily inoculations of freshly grown organisms. The embedded silver complex demonstrated a faster kill rate than Silvadene^®^ and AgNO_3_, as well as having a prolonged release of the active silver species.

Further investigations of biologically active Ag(I)-NHC complexes led to the development of a series of xanthine derivatives, beginning with complex **4** (Figure 2) [28]. The MIC of complex **4** was tested against a variety of Gram-positive and Gram-negative bacteria, including *E. Coli* J53 strains, with and without the silver resistant plasmid pMG101. Complex **4** showed good activity against all pathogens tested with MIC values ranging from 1 to 8 μg/mL, with the exception of the *E. Coli* J53 strain with silver resistance (>5000 μg/mL). This further demonstrated that the bioactivity was primarily due to the Ag(I) cation. Complex **4** also showed antimicrobial activity against several strains of *Burkholderia cepacia* (*B. cepacia*), a highly resistant respiratory pathogen found in patients with cystic fibrosis. After the initial success of **4**, a series of Ag(I)-NHC complexes were synthesized and their bioactivity was evaluated [29,30,31]. Of note, complex **5** showed efficacy comparable to **4**, while also demonstrating increased water solubility. Complexes **4** and **5** were also found to be effective against *B. cepacia* in vivo through nebulization into the lungs of mice [32]. Of the mice that were infected with *P. aeruginosa* and subsequently treated with nebulized **4**, 88% (22/25) survived after 72 h, whereas 62% (16/26) of water treated mice survived. In a separate study, of all the *P. aeruginosa* infected mice that were treated with nebulized **4**, 83% (5/6) survived compared to 17% (1/6) of water treated mice [33]. Several of the Ag(I)-NHC complexes were tested against MRSA, as well as the potential bioterrorism agents *Burkholderia pseudomallei*, *Burkholderia mallei*, *Bacillus anthracis*, and *Yersinia pestis* [34]. Compounds **6**–**9** (Figure 2) demonstrated MIC values of <6 μg/mL when tested against *B. pseudomallei* and *B. mallei*. Compounds **4** and **6** were also tested against two attenuated strains of *Y. pestis*, YP1-1 and YP8-1, and showed MIC values of 1 μg/mL.

**Figure 3:**
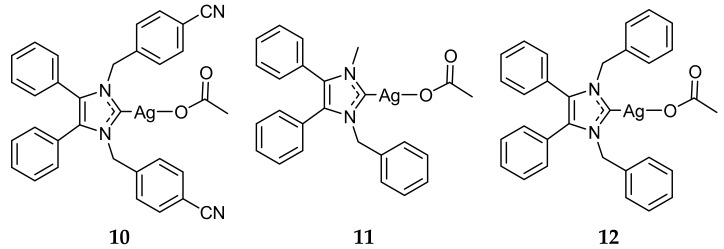
Structures of Ag(I)-NHC complexes synthesized by Tacke and coworkers [42,43].

Once the utility of Ag(I)-NHC complexes was established, the synthesis of these compounds garnered much interest abroad. Tacke and coworkers synthesized a vast library of Ag(I)-NHC acetate complexes, the majority containing large lipophilic substituents [35,36,37,38,39,40,41]. The antibacterial properties of many of these complexes were tested against *E. coli* and *S. aureus* using the qualitative Kirby-Bauer disk-diffusion method. The majority of the Ag(I)-NHC compounds exhibited low to medium activity (4–7 mm of clearance) against both Gram-positive and Gram-negative bacteria, while their NHC precursors showed significantly lower activity, again indicating the utility of Ag(I) ions in antimicrobial Ag(I)-NHC complexes. Several compounds, including **10** and **11** (Figure 3), showed high antibacterial activity with areas of clearance of 10–12 mm. The improved antibacterial properties were attributed in part to the lipophilicity of these complexes, which allowed for increased penetration through the lipid membrane. This led to the development of the lead antimicrobial compound for the Tacke research group, complex **12** (Figure 3) [42,43]. While **12** exhibited medium activity against *E. coli*, showing 7 mm area of clearance, the complex was highly active against *S. aureus* with 15 mm of clearance. Complex **12** was further tested quantitatively against several Gram-positive and Gram-negative bacterial strains and was found to have MIC values ranging from 3.13 to 20 μg/mL, including being active against MRSA with an MIC value of 12.5 μg/mL [44]. The antimicrobial activity of **12** was also evaluated in vivo using *Galleria mellonella* larvae. Larvae inoculated with *S. aureus* and *Candida albicans* and subsequently treated with **12** showed an increased survival rate over those untreated [45].

**Figure 4:**
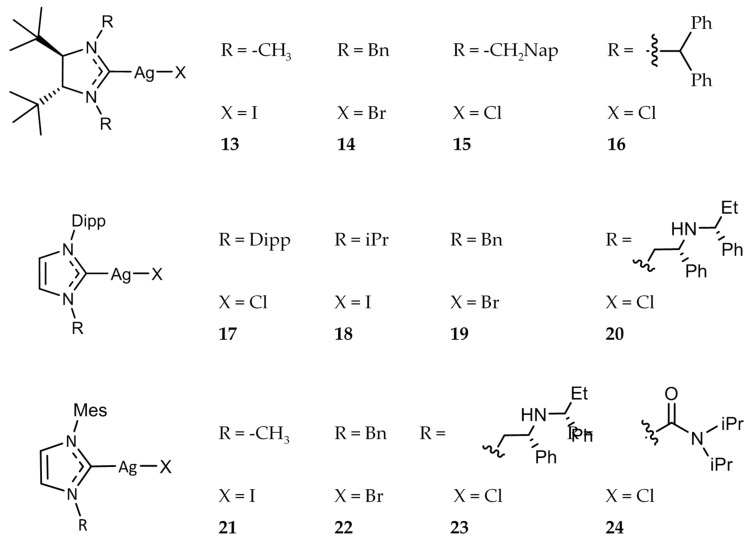
Structures of Ag(I)-NHC complexes from Roland and Jolivalt [46].

Roland et al. synthesized a series of Ag(I)-NHC halide complexes (Figure 4) and evaluated their antibacterial activity [46]. The compounds were tested against *E. coli* and *S. aureus* as well as several resistant strains including *S. aureus* NorA, a ciprofloxacin resistant strain that overexpresses the multidrug efflux pump NorA, and *S. aureus* MsrA, which contains the plasmid pUL5054 that gives rise to erythromycin resistance. Complexes **13**–**16** and **18**–**24** (Figure 4) demonstrated high activity against *E. coli* and *S. aureus* with MIC values ranging from 4 μg/mL to 32 μg/mL. Complex **17** showed no significant activity against *E. coli* with an MIC value of >128 μg/mL. All of the complexes tested, including **17**, were found to have significant activity against both resistant strains as well, with a range of MIC values from 1 μg/mL to 32 μg/mL.

**Figure 5:**
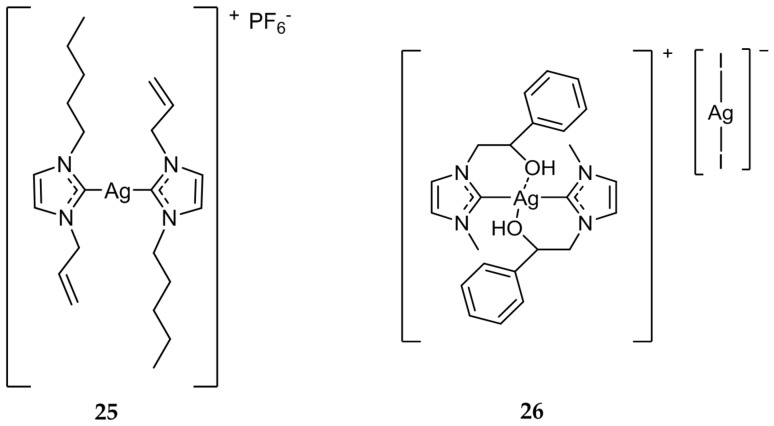
Structure of bis-NHC complexes from Haque (**25**) and Napoli (**26**) [51,52].

While the majority of the Ag(I)-NHC compounds that have been evaluated are mono-NHC systems, there have been many bis-NHC systems developed as well [47,48,49]. In 2014, Haque et al. synthesized a series of bis-NHC systems and evaluated their antibacterial properties and subsequent nuclease activity [50,51]. Of the compounds synthesized and tested, **25** (Figure 5) was shown to not only be the most effective antibacterial complex (MIC = 12.5 μg/mL), but also was the most efficient in promoting the cleavage of nucleic acids. This correlation could give some indication about the mechanism of action of Ag(I)-NHC antimicrobials.

Napoli et al. developed several compounds that contained an alcohol group on an alkyl substituent of one of the two nitrogens of the NHC core [52]. Compound **26** (Figure 5) inhibited the growth of both *E. coli* and *B. subtilis* at a concentration of 5 μg/mL, the highest activity of any bis-NHC complex that was evaluated in the study. Compound **26** was also shown to be stable to hydrolysis, which could lead to slow release of bioactive Ag(I) at the wound site, further showing the utility of Ag(I)-NHC compounds as antimicrobial agents.

**Figure 6:**
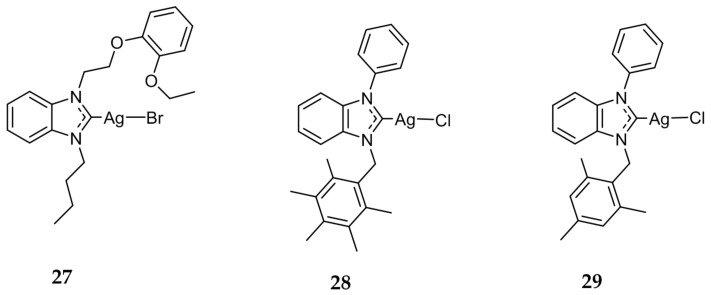
Structures of benzimidazole Ag(I)-NHC complexes from Özdemir (**27**) [54] and Gök (**28** and **29**) [55].

A series of silver benzimidazole compounds were synthesized by Özdemir et al. and tested against several bacterial strains including *Enterococcus faecalis*, *S. aureus*, *E. coli*, and *P. aeruginosa* [53,54]. While none of the compounds outperformed the standard antimicrobials ampicillin or ciprofloxacin, it was determined that the presence of electron-donating and bulky substituents attached to the nitrogen of the benzimidazole ring increases the antimicrobial activity of the compound. The most lipophilic compound, **27** (Figure 6), was found to be the most active with MIC values ranging from 6.25 μg/mL to 12.5 μg/mL against the series of bacterial strains.

In a separate study performed by Gök et al. in 2014, phenyl-substituted benzimidazole silver complexes were tested for their antibacterial properties against several Gram-positive and Gram-negative bacterial strains [55]. While the Ag(I)-NHC compounds showed increased antimicrobial activity when compared the NHC precursors, they displayed lower activity when compared to the standard tetracycline. Similarly to the previous study conducted by Özdemir et al. the most lipophilic compounds **28** and **29** (Figure 6) were found to be the most active.

## 3. Antitumor Activity of Silver NHC Complexes

With the serendipitous discovery of cisplatin in the late 1960s, the landscape of cancer chemotherapy was forever changed [56,57]. A research field that was previously dominated by organic compounds and natural products shifted towards the inclusion of metallo-pharmaceuticals. With platinum being at the forefront of research, many other platinum-based compounds were approved for antitumor therapy including carboplatin and oxaliplatin [58]. While the curative effects are well documented, the drawbacks of platinum-based pharmaceuticals are numerous. Many cancer types are not susceptible to platinum drugs and there are many toxic side effects, including gastrointestinal and hematological toxicity [59]. Additionally, many cancers have either acquired or intrinsic resistance to cisplatin and other platinating agents [60]. Because of this, current anticancer research has been devoted to the discovery of novel transition metal compounds. Due to their synthetic versatility and strong, stable metal-ligand bond, NHC-metal complexes have become a vastly increasing class of anticancer agents. While silver was originally investigated because of its advantageous antimicrobial activity, there has been a recent interest in its anticancer properties.

Youngs and coworkers were the first to report the anticancer activity of Ag(I)-NHC compounds with a series of 4,5-dicholorimidazole based Ag(I)-NHC compounds [61]. Compounds **6**–**8** (Figure 2), previously discussed for their antimicrobial properties, were evaluated for their in vitro antitumor activity against the cancer cell lines OVCAR-3 (ovarian), MB157 (breast), and HeLa (cervical). The concentrations at which the compound inhibits 50% of cell growth (IC_50_) as compared to a control were compared to both cisplatin and AgNO_3_. Cisplatin was determined to be the most effective against the ovarian cancer cell line with an IC_50_ value of 12 μM, compared to 20–25 μM for the silver complexes. Compounds **6**–**8** were also determined to be ineffective against the HeLa cell line (>200 μM). However, the silver compounds were most active against MB157, having IC_50_ values of 8 μM, 20 μM, and 10 μM for **6**–**8** respectively, compared to an IC_50_ value of 25 μM for cisplatin.

With the in vitro success of these compounds, an in vivo xenograft model was developed utilizing OVCAR-3 and **6**. OVCAR-3 cells were injected subcutaneously into the back of nude mice. After tumor growth became visible, **6** was injected at the tumor site. The mice were necropsied to determine the overall effect of **6** on the tumors as well as healthy organs. According to the pathological studies, significant tumor cell death was observed while no ill-effects to the major organs of the mice were detected.

**Figure 7:**
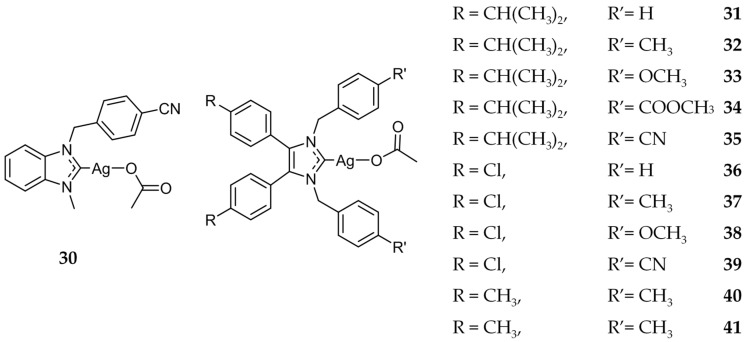
Structures of Ag(I)-NHC complexes from Tacke and coworkers [43,62,63].

After this initial success of Ag(I)-NHCs, Tacke and coworkers synthesized a series of cyanobenzyl-NHC silver complexes and evaluated their anticancer activity [43,62,63]. The in vitro cytotoxicity of these compounds were evaluated against the human cancerous renal cell line, Caki-1. From these studies, compound **30** (Figure 7) was shown to have an IC_50_ value of 1.2 ± 0.6 μM, an approximately three-fold increase compared to cisplatin (3.3 μM). It was further shown that **30** was effective in vitro against platinum-resistant cell lines UKF-NB-3 (neuroblastoma) and HCT8 (colon), as well as paclitaxel-resistant cell line PC-3 (prostate) [64]. While these in vitro results were encouraging, in vivo testing of **30** showed no tumor reduction in tumor bearing mice.

Tacke and coworkers also investigated the potential of benzyl bearing NHC compounds as antitumor agents, (compounds **31**–**41**, Figure 7), similar to **12** (Figure 3) discussed previously for its antimicrobial activity [40,41]. Of these compounds, **41** demonstrated the highest in vitro activity with IC_50_ values of 0.51 ± 0.07 μM and 1.4 ± 0.1 μM against Caki-1 and MCF-7 (breast) cell lines respectively. Additionally, the NHC precursor to **41** was also determined to be highly active against Caki-1 (IC_50_ = 4.8 ± 0.3 μM), indicating a synergistic effect with the NHC ligand and the silver component.

**Figure 8:**
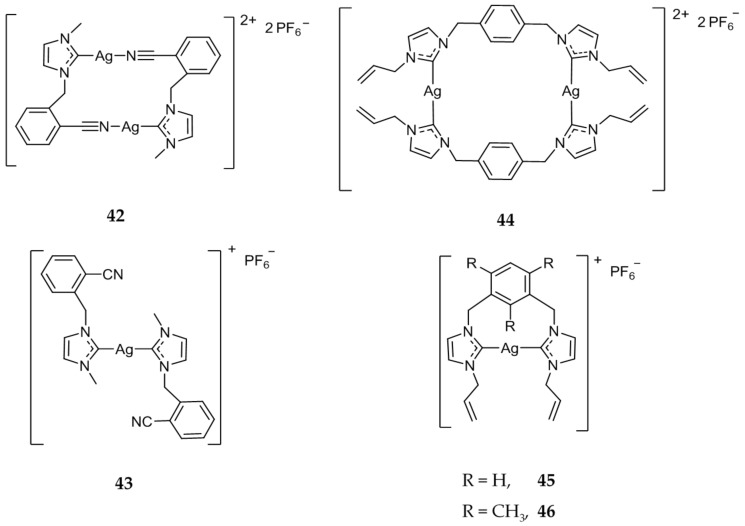
Mono- and dinuclear Ag(I)-NHC complexes from Haque and coworkers [70,71].

An extensive library of both mono- and dinuclear silver carbene compounds were synthesized and the anticancer efficacy was compared with several standard treatments [47,48,49,50,65,66,67,68,69]. The in vitro activity of **42** (Figure 8), a dinuclear Ag(I)-NHC compound, showed increased activity (IC_50_ = 1.7 μM) when compared to the mononuclear compound **43** (Figure 8) (IC_50_ = 6.0 μM) against HCT 116 (colon), attributed to the possible increased stability and subsequent slowed release of Ag(I) ions [70]. Compounds **44**–**46** (Figure 8) were also tested against HCT 116 cell line and showed high activity with IC_50_ values of 0.9, 1.3, and 1.1 μM respectively, much more cytotoxic than the standard drug 5-fluorouracil (5-FU) (IC_50_ = 5 μM) [71].

**Figure 9:**
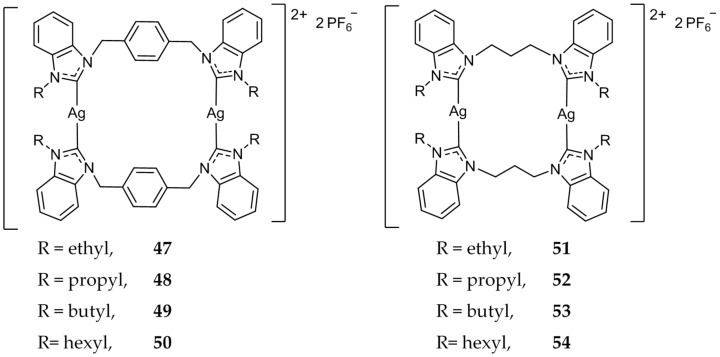
Dinuclear benzimidazole Ag(I)-NHC complexes from Haque and coworkers [72,73,74,75,76,77].

These xylyl-linked systems were further studied and **48** and **49** (Figure 9) showed an increased potency, with IC_50_ values of 0.4 and 0.01 μM against HCT 116, compared to 19.2 μM for 5-FU [72]. Additionally, several compounds were synthesized with varying lengths of alkyl chains as terminal *N*-substitutions [73,74,75]. Notably, **50** (Figure 9) was evaluated for cytotoxicity against HCT 116 as well as normal fibroblast (CCD-18Co) cells [76]. While **50** showed potent cytotoxicity against the cancerous cell line HCT 116 (IC_50_ = 1.7 μM), it did not show any activity (IC_50_ > 200 μM) against normal colon fibroblasts (CCD-18Co) cells. Compound **50** was also found to induce pro-apoptotic changes in the cellular nucleus via the Hoechst 33342 assay. It was further determined that **50** induced cleavage of caspase-3/7 in HCT 116 cells via FAM-FLICA caspase assay. Most recently, Haque et al. tested the efficacy of propylene linked bis-benzimidazole compounds **51**–**54** (Figure 9) against MCF-7 [77]. All four compounds showed comparable activity (IC_50_ = 7 ± 1 μM–18 ± 3 μM) compared to the standard drug Tamoxifen (IC_50_ = 11 ± 2 μM). The anticancer results showed that all dinuclear Ag(I)-NHC complex were active while their corresponding NHC salts were not.

**Figure 10:**
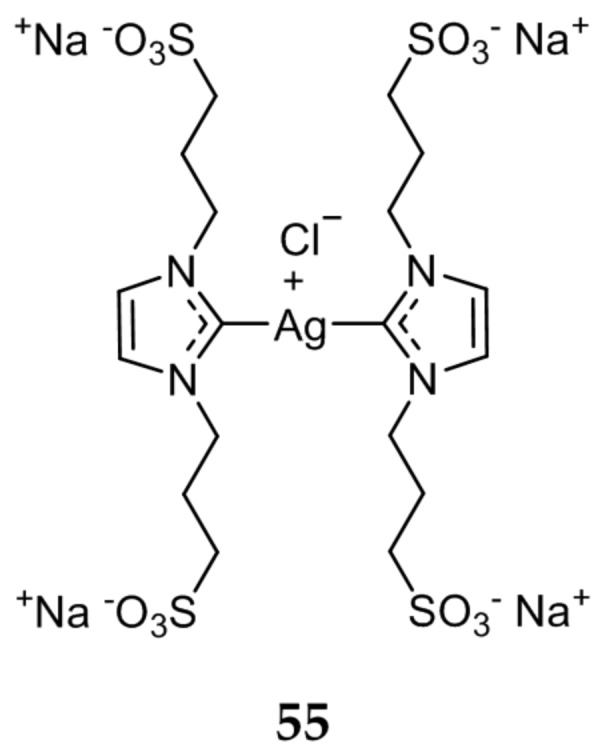
Hydrophilic sulfonated Ag(I)-NHC from Gandin et al. [78].

Several water soluble Ag(I)-NHC compounds were synthesized by Gandin et al. and their antiproliferative activity was evaluated [78]. The sulfonate functionalized Ag(I)-NHC **55** (Figure 10), was determined to be the most promising compound. The in vitro antitumor activity of **55** was tested against the human tumor cells lines A549, HCT-15, MCF-7, A431 (cervical), and A375 (melanoma) and had comparable activity to cisplatin. Compound **55** had an average IC_50_ value of 11.63 μM against all five cell lines, compared to an average IC_50_ of 8.50 μM for cisplatin. Compound **55** was also shown to be effective against cisplatin resistant cells (C13*), with an IC_50_ value of 11.26 ± 2.11 μM, nearly two fold lower than cisplatin (IC_50_ = 22.54 ± 2.16 μM). Further mechanistic studies revealed a strong correlation between the high in vitro antiproliferative activity of **55**, and its ability to inhibit thioredoxin reductase (TrxR). This inhibition of TrxR led to the activation of the ASK-1 pathway, leading to apoptotic cell death.

**Figure 11:**
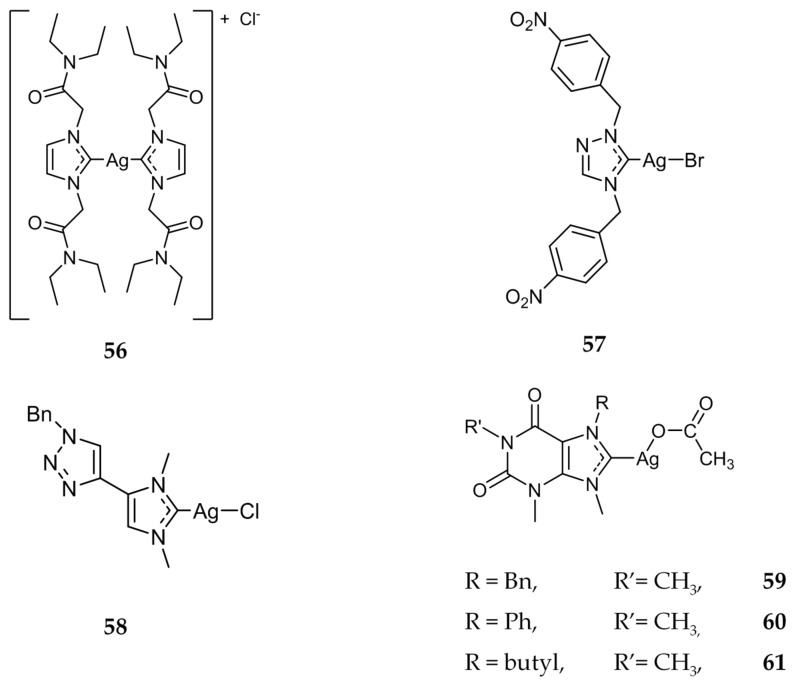
Structures of Ag(I)-NHC complexes from Santini (**56** and **57**) [79,80,81], Bellemin-Laponnaz and Tubaro (**58**) [82], and Willans (**59**–**61**) [83].

Several other research groups also synthesized, characterized, and investigated the cytotoxic properties of bis-NHC compounds [79,80]. The most promising derivative synthesized by Santini, Dias and coworkers was compound **56** (Figure 11). The IC_50_ values for **56** against HCT-15 (14.11 ± 2.11 μM) and A549 (lung; IC_50_ = 16.23 ± 2.31 μM) cell lines were comparable to that of cisplatin with IC_50_ values of 15.25 ± 2.31 μM and 13.10 ± 1.23 μM respectively. Compound **56** was also subjected to in vitro testing to evaluate its ability to inhibit TrxR. It was found that **56** was able to decrease the TrxR activity by ~80% at a low concentration of 90 nM, further indicating this enzyme as a target for Ag(I)-NHC compounds.

Santini, Gandin, and coworkers also developed a series of triazolium NHCs, including compound **57** (Figure 11) [81]. When compared to cisplatin, **57** displayed higher activity against several cancerous cell lines, including HCT-15, BxPC3 (pancreatic), A549 (lung), as well as C13*, a cisplatin-resistant ovarian cell line. Similarly to Ag(I)-NHC compounds discussed previously, **57** was also shown to inhibit TrxR. After a 15 min incubation, TrxR was inhibited by ~46% at the IC_50_ of 7.2 nM. In 2017, another triazole substituted Ag(I)-NHC complex was synthesized, compound **58** (Figure 11) [82]. This compound showed good activity against all three cancer cell lines that were tested; HCT116 (IC_50_ = 1.38 ± 0.36 μM), MCF7 (IC_50_ = 2.0 ± 0.2 μM), and PC3 (IC_50_ = 1.38 ± 0.36 μM). The Ag(I)-NHC compound out performed cisplatin against all three cell lines.

Several xanthine derivatives were synthesized and tested by Willans, Phillips, and coworkers [83]. Compounds **59**–**61** (Figure 11), as well as compounds **4** and **5**, previously discussed for their antimicrobial properties, were evaluated against several cancer cell lines and showed moderate activity, with all IC_50_ values being in the micromolar range . All the compounds tested were less active than cisplatin. The most cytotoxic silver complex was **60**, with the NHC ligand having a phenyl group as a substituent. This phenomenon is attributed by the authors to the phenyl group having a stabilizing effect on the silver NHC bond, slowing the release rate of the silver ions.

**Figure 12:**
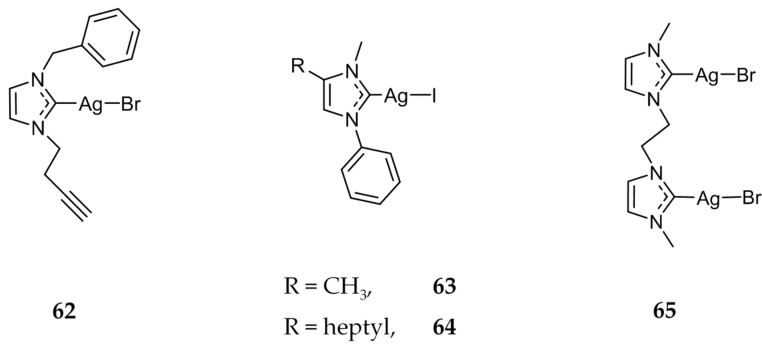
Structures of Ag(I)-NHC complexes from Garner et al. (**62**) [84], Bjørsvik et al. (**63** and **64**) [85], and Sanchez et al. (**65**) [86].

Three alkyne functionalized Ag(I)-NHC compounds were synthesized and evaluated for their applications in biomolecule conjugation [84]. The cytotoxicity of **62** (Figure 12) was found to be in the micromolar range against a variety of human cancer cell lines including DLD-1 (colon; IC_50_ = 6.8 ± 0.8 μM), Hep-G2 (liver; IC_50_ = 6.9 ± 0.7 μM), MCF-7 (IC_50_ =17.1 ± 0.4 μM), and HEK (liver, IC_50_ = 22.6 ± 0.9 μM).

The effect of varying the length of an aliphatic chain substituted on the 4-position of the imidazole was also studied for mononuclear **63** and **64** (Figure 12). Bjørsvik et al. compared the activity of **63** and **64** against two leukemia cell lines HL60 and MOLM-13 [85]. Compound **64** was much more active against both cell lines showing IC_50_ values of 14 μM against HL60 and 27 μM against MOLM-13, compared to **63** with IC_50_ concentrations of 78 μM and 123 μM respectively. For both compounds, the authors determined cell death to be apoptotic via Hoechst 33342 and Annexin V staining.

Compound **65** (Figure 12) was synthesized and is proposed to interact with DNA mainly through non-covalent interactions as π-π stacking contacts, inducing a conformational change in the structure of DNA [86]. The Ag(I)-NHC complex showed similar activity to cisplatin against prostate (PC3) and colon (HT29) cancerous cell lines.

**Figure 13:**
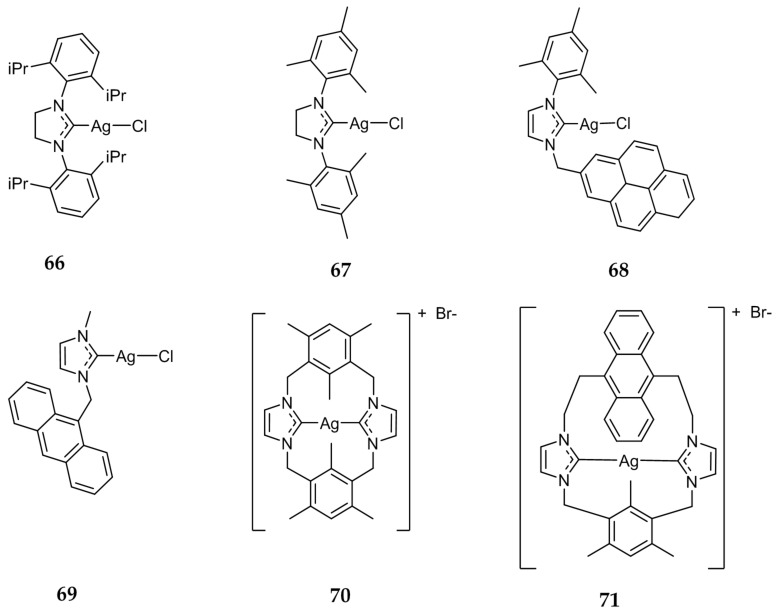
Structures of Ag(I)-NHC complexes from Eloy et al. (**66**–**68**) [87], Citta et al. (**69**) [88], and Li et al. (**70** and **71**) [89].

Eloy et al. evaluated a series of Ag(I)-NHC complexes for their antitumor activity and explored their mechanism of action [87]. Compounds **66** and **67** (Figure 13), as well as compounds **13**–**24** (Figure 4), previously discussed for their antibacterial activity, were tested against a variety of cancer cell lines, including drug-resistant cells, with some compounds exhibiting IC_50_ values in the nanomolar range. Compounds **17** and **66** were found to be the most active, having a range of IC_50_ values of 28 ± 1–75 ± 15 nm against HCT 116, MCF-7, and HL60 cell lines. These compounds demonstrated a drastic increase in activity as compared to cisplatin (IC_50_ = 2.7–5.9 μM). Compounds **17**, **66**, and **67** were also shown to induce cell death via apoptosis without the involvement of necrosis against HL60 cells. Further studies showed that the apoptotic cell death caused by these Ag(I)-NHC compounds was likely due to the depolarization of the mitochondria membrane potential (∆Ψ_m_), not associated with the overproduction of reactive oxygen species (ROS) nor with caspase-3 activation. Cell death in HL60 cells by **17**, **66**, and **67** was shown to be caspase-independent apoptosis, triggered by translocation of apoptosis-inducing factor and caspase-12 into the nuclear compartment. Mitochondria was further indicated as a target for Ag(I)-NHC complexes by Eloy et al. by utilizing the fluorescent compound **68**.

In 2013, the fluorescent Ag(I)-NHC compound **69** (Figure 13) was synthesized and evaluated by Citta et al. [88]. Compound **69** showed IC_50_ values in the low micromolar range against cisplatin sensitive A2780S (IC_50_ = 3.26 ± 0.15 μM), cisplatin resistant A2780R (IC_50_ = 3.73 ± 0.18 μM), and non-tumorigenic kidney cell line HEK-293T (IC_50_ = 6.36 ± 0.25 μM). Compound **69** showed an increased selectivity for cancer cells compared to its gold analogue. Further studies demonstrated that **69** inhibited rat cytosolic and mitochondrial thioredoxin reductases as well as the Se-free enzyme glutathione reductase. Fluorescence microscopy studies showed that **69** was distributed intracellularly in tumor cells where they are able to reach the nuclear compartment.

More recently, Li et al. evaluated the antitumor properties of **70** and **71** (Figure 13) [89]. These compounds displayed superior or comparable activity to cisplatin against the cancer cell lines HeLa, A549, MDA-MB-231 (breast), as well as cisplatin resistant A549R. Compounds **70** and **71** also demonstrated lower cytotoxicity against normal liver LO2 cells as compared to cisplatin. Similarly to studies conducted by Eloy et al. **70** and **71** were found to induce apoptosis through modification of ∆Ψ_m_, independent of ROS- and caspase pathways.

**Figure 14:**
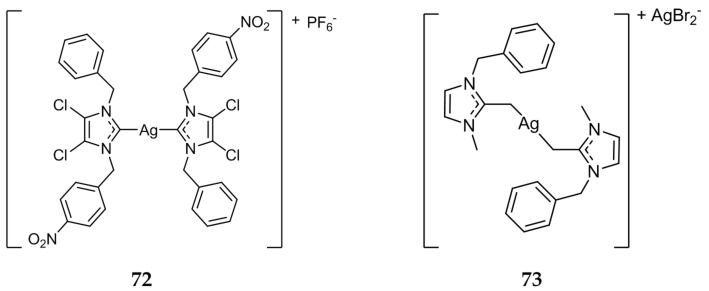
Structures of Ag(I)-NHC complexes from Sahanini et al. (**72**) [90] and Allison et al. (**73**) [91].

A series of nitrobenzyl substituted mono- and bis-NHC complexes were synthesized and tested against MCF-7 cell line via MTT assay [90]. All compounds tested exhibited IC_50_ values in the nanomolar range, the bis-NHC complex **72** (Figure 14) showing the highest anticancer potential with an IC_50_ value of 10.39 nM. The authors indicated, with the exception of **72**, mono-NHC compounds displayed better activity than the bis-NHC analogues in this study.

In 2017, Allison et al. evaluated the biological activity of compound **73** [91]. Compound **73** (Figure 14) was more active in vitro against A2780, an ovarian cancer cell line, with an IC_50_ value of 0.44 ± 0.15 μM when compared to cisplatin (IC_50_ = 0.73 ± 0.30 μM). A significantly increased activity was also demonstrated against the cisplatin resistant strain A2780*cis*/CP70, with an IC_50_ value of 0.09 ± 0.01 μM for compound **73** compared to an IC_50_ of 6.07 ± 1.78 μM for cisplatin. Compound **73** was also found to be a very potent inhibitor of TrxR (IC_50_ = 2.39 ± 0.59 nM) and was shown to inhibit topoisomerase I in cell-free assays, with complete inhibition at 0.16 μM. The DNA repair enzyme PARP-1 was also inhibited by **73** in vitro, with an IC_50_ value of 32 ± 7.6 nM, significantly more potent than the known PARP-1 inhibitor NU1025 (IC_50_ = 2.4 ± 0.27 μM).

## 4. Antitumor Activity of *N*-Heterocyclic Carbene Silver Complexes as Compared to Their Precursor Imidazolium Salts

While silver is known to have biological activity, less is known about imidazolium salts. However, by effectively delivering the silver, an imidazolium salt is also released and the therapeutic effect of it should also be of interest. Not only can imidazolium salts be tuned to release silver at the optimal time, they can also be modified so as to better act as anticancer agents themselves. Each type of functional group placed in a different position of the imidazole ring changes the activity of the imidazole and can alter the release of Ag(I) ions.

**Figure 15:**
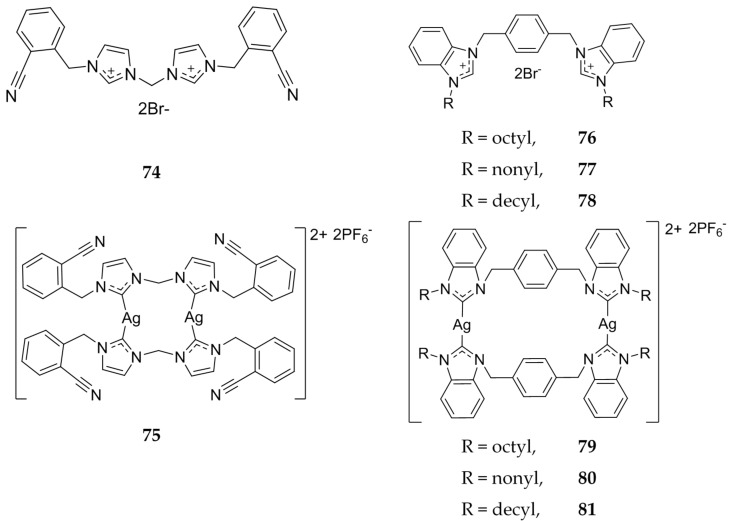
Structures of imidazolium salts and benzimidazolium salts and corresponding Ag(I)-NHCs from Haque and coworkers [75].

In 2013, Haque et al. evaluated cyanobenzyl bis-imidazolium salts as both the ligand and dinuclear Ag(I)-NHCs (Figure 15) using the MTS assay [75]. Against the human immortalized myelogenous leukemia cell line K562, compound **74** showed no activity (IC_50_ >200 μM) whereas **75** was shown to have comparable activity to that of 5-FU with IC_50_ values of 43.7 μM and 35.9 μM, respectively. In addition, *para*-xylyl linked benzimidazole ligands, **76**–**78**, were compared to the dinuclear silver complexes, **79**–**81** (Figure 15). Overall, the benzimidazole ligands and silver complexes were more effective against the K562 cell line than the imidazole compounds, with an increase in alkyl chain length from octyl to nonyl to decyl, corresponding to better activity. IC_50_ values of the ligands alone were 34.9 μM, 8.9 μM, and 9.0 μM, with respect to an increase in alkyl length. The dinuclear silver complexes corresponding to the ligands were shown to be more active with IC_50_ values of 6.57 μM, 3.67 μM, and 3.37 μM, respectively. From the IC_50_ values it is clear that the silver complexes are more active than the ligand alone. However, in comparison to 5-FU, the benzimidazole ligands are more effective against leukemia.

**Figure 16:**
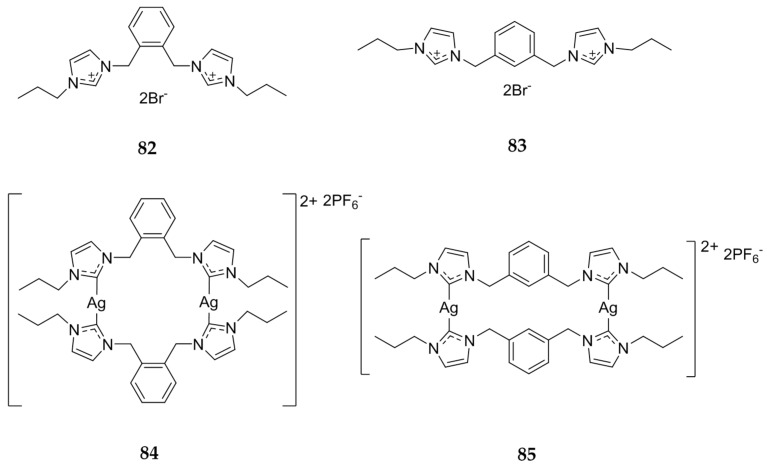
Structures of imidazolium salts and corresponding Ag(I)-NHCs from Haque and coworkers [92].

Haque et al. published another paper in 2013 investigating bis-imidazolium salts with either an *ortho-* or *meta*-xylyl linkage [92]. Again, the precursor imidazolium salt was directly compared to the Ag(I)-NHC complex using the MTT assay on both HCT 116 and MCF-7 cell lines. The bis-imidazolium salts **82** and **83** (Figure 16) were inactive against the HCT 116 cells (IC_50_ > 200 μM) while a slight toxicity against the MCF-7 cells was observed (IC_50_ = 158 to >200 μM). The silver complexes **84** and **85** (Figure 16) showed a significantly greater cytotoxic effect with the *meta*-xylyl linked compound, **85**, which displayed IC_50_ values of 5.6 μM for HCT 116 and 1.12 μM for MCF-7. This data shows that alone, the bis-benzimidazoles are much more effective than the bis-imidazoles and the silver complexes are even more effective, especially when a larger cage has been achieved with the *meta*-xylyl linker, as outlined by the authors.

**Figure 17:**
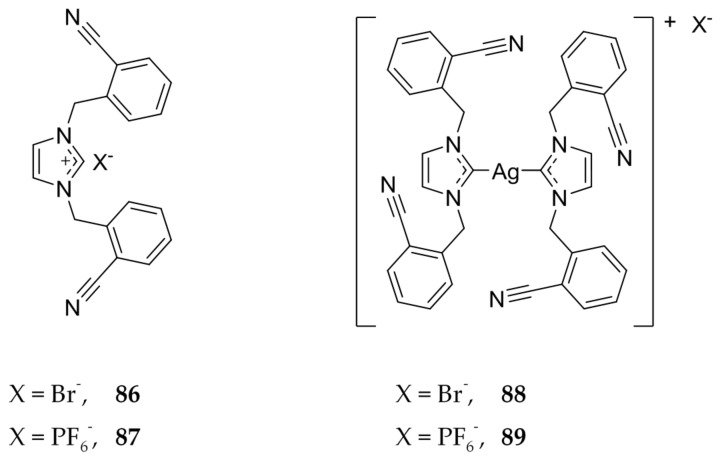
Structures of imidazolium salts and corresponding Ag(I)-NHC complexes from Zulikha et al. [70].

Zulikha et al. also found a ligand that had greater cytotoxicity than 5-FU against HCT 116 cells using the MTT assay (Figure 17) [70]. Interestingly, the active compound, **86** (IC_50_ = 5.0 ± 0.3 μM), differs from an inactive counterpart, **87** (IC_50_ > 200 μM), in only the counterion associated with the ligand. A similar trend is seen with the corresponding Ag(I)-NHCs, **88** and **89** (Figure 17), with IC_50_ values of 1.7 ± 0.2 μM and 27.2 ± 1.1 μM, respectively. Both **86** and **87** were found to have better cytotoxicity than the control, 5-FU. The authors suggest that the PF_6_^−^ anion contributes to the stability of the silver NHC bond and thus does not allow for a quick release of silver, leading to less cytotoxicity.

**Figure 18:**
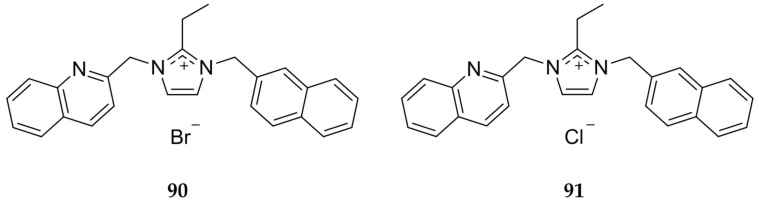
Structures of imidazolium salts synthesized by Youngs and coworkers [93].

Not only does the anion effect cytotoxicity of imidazolium salts and corresponding Ag(I)-NHCs, it can also alter the solubility as displayed by Youngs and coworkers [93]. The bromide and chloride imidazolium salt, **90** and **91** respectively (Figure 18), were directly compared and it was determined that solubility of the chloride derivative (>10 mM) was at least 2-fold higher than with a bromide anion (~5 mM). This shows that while functional groups substituted on the imidazole core can change activity of both the imidazolium salt and the resulting Ag(I)-NHC, the anion selection is important as well for both stability and solubility.

**Figure 19:**
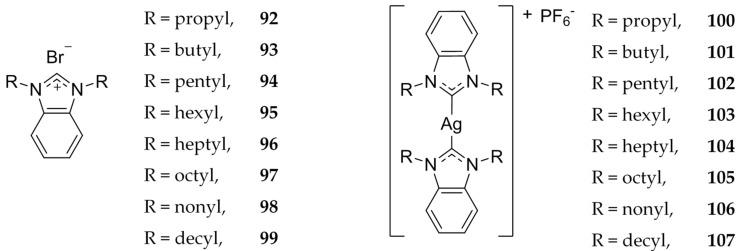
Structure of monobenzimidazolium salts and corresponding bis-imidazolium Ag(I)-NHCs from Haque and coworkers [94].

In 2017, Fatima et al. evaluated a group of benzimidazole compounds with varied alkyl chains off of the nitrogen positions of the ring (Figure 19) [94]. Cytotoxicity was determined by the MTT assay on HCT 116 cells. It was found that increasing the alkyl chain length to 6 carbons or more marked a significant decrease in IC_50_ value for both the ligand and the Ag(I)-NHC complex. The ligand alone has an IC_50_ range between 27.3 ± 0.63 and 31.8 ±3.84 μM with less than 6 carbons in the alkyl chain and a range of 1.4 ± 0.15–7.5 ± 2.22 μM with 6 or more in the alkyl chain. Similarly, the silver complex had IC_50_ ranges of 13.2 ± 1.50–26.8 ± 2.30 μM and 0.02 ± 0.02–3.9 ± 0.62 μM, respectively. In fact, all compounds with 6 or more carbons in the alkyl chain length exhibited greater cytotoxicity than 5-FU (IC_50_ = 10.2 μM). The silver NHCs in this case have lower IC_50_ values than the ligands themselves. Authors attribute this to the release of silver. This further establishes the importance of the relationship between lipophilicity and cytotoxicity, as demonstrated in previous studies [95,96,97,98].

With the large variety of modifications that can be done on imidazolium salts, compounds to treat many different kinds of cancer can be developed. As displayed above, a delicate balance between lipophilicity, functional groups and associated anion must be found for the best activity of both the imidazolium salt alone and the corresponding silver NHC complexes.

## 5. Conclusions

Since the beginning of their isolation by Arduengo, NHCs have proved to be a versatile area of research. Their ability to bind different types of metals, as well as the multitude of chemical modifications that exist, makes them useful in many areas of scientific study, including new antibacterial and chemotherapeutic compounds. Specifically, Ag(I)-NHCs have shown promising outcomes in both of these areas.

While the antibacterial properties of silver have been well established, NHCs serve as an efficient way to deliver Ag(I) where needed. Ag(I)-NHCs have been found to be active against both Gram-positive and Gram-negative species, while also being potent enough to act against bioterrorism agents including *Burkholderia pseudomallei* and *Yersinia pestis*. Not only have Ag(I)-NHCs shown promise with in vitro studies, some have also increased the survival rate of infected *Galleria mellonella* larvae and infected mice when tested in vivo.

Recently, Ag(I)-NHCs have also been developed and utilized for the purpose of potential chemotherapeutic drugs as well. Using in vitro assays, Ag(I)-NHCs show toxicity comparable to, or better than, current chemotherapeutics such as cisplatin and 5-fluorouracil in a variety of cancer cell types. Functional groups on the NHC core have been shown to greatly affect toxicity, with lipophilicity and counterion playing an important role. While the mechanism of action of Ag(I)-NHC chemotherapeutics is still unclear, several possible biological targets have been identified. However, further research is still required to fully elucidate the mechanism of action of Ag(I)-NHC complexes.
